# *Shigella* Mediated Depletion of Macrophages in a Murine Breast Cancer Model Is Associated with Tumor Regression

**DOI:** 10.1371/journal.pone.0009572

**Published:** 2010-03-08

**Authors:** Katharina Galmbacher, Martin Heisig, Christian Hotz, Joerg Wischhusen, Antoine Galmiche, Birgit Bergmann, Ivaylo Gentschev, Werner Goebel, Ulf R. Rapp, Joachim Fensterle

**Affiliations:** 1 Institut für Medizinische Strahlenkunde und Zellforschung, Universität Würzburg, Würzburg, Germany; 2 Institut für Mikrobiologie, Biozentrum, Universität Würzburg, Würzburg, Germany; 3 Universitätsfrauenklinik Würzburg, Würzburg, Germany; Technical University Munich, Germany

## Abstract

A tumor promoting role of macrophages has been described for a transgenic murine breast cancer model. In this model tumor-associated macrophages (TAMs) represent a major component of the leukocytic infiltrate and are associated with tumor progression. *Shigella flexneri* is a bacterial pathogen known to specificly induce apotosis in macrophages. To evaluate whether *Shigella-*induced removal of macrophages may be sufficient for achieving tumor regression we have developed an attenuated strain of *S. flexneri* (M90T*ΔaroA*) and infected tumor bearing mice. Two mouse models were employed, xenotransplantation of a murine breast cancer cell line and spontanous breast cancer development in MMTV-HER2 transgenic mice. Quantitative analysis of bacterial tumor targeting demonstrated that attenuated, invasive *Shigella flexneri* primarily infected TAMs after systemic administration. A single i.v. injection of invasive M90T*ΔaroA* resulted in caspase-1 dependent apoptosis of TAMs followed by a 74% reduction in tumors of transgenic MMTV-HER-2 mice 7 days post infection. TAM depletion was sustained and associated with complete tumor regression.

These data support TAMs as useful targets for antitumor therapy and highlight attenuated bacterial pathogens as potential tools.

## Introduction

Ever since the American surgeon Coley described streptococcal infection as a potential cure of cancer [1], other bacteria have been explored and were shown to infiltrate, replicate and accumulate in tumors [2], [3]. For some extracellular bacteria, such as genetically modified obligate anaerobe *Clostridium novyi*, an anti-tumor effect was observed [4]. Other extracellular bacteria such as *Escherichia coli* accumulated in tumor tissue, induced some inflammatory responses, but failed to confer protection [5]. Facultative intracellular bacteria such as *Salmonella* have also been assessed for tumor therapy and their intratumoral accumulation was studied using different technologies, albeit beyond cellular resolution [6], [7], [8], [9]. In most syngenic experimental models the therapeutic effect was moderate, whereas in xenograft models a more pronounced effect was described [7], [9], [10], [11], [12]. It was speculated that induction of an inflammatory response was mediating the anti-tumor effect.

In contrast to extracellular bacteria, intracellular bacteria can deliver DNA into eukaryotic cells [13]. Therefore, intracellular bacteria could be employed to deliver toxins or prodrug converting enzymes directly into tumor cells. Yet, no quantitative information on the distribution of intracellular bacteria in different stromal versus tumor cells is available even though such data are key to the design of effective therapeutic regimens [14].

Tumors consist of a complex mixture of transformed cells and stroma cells [15]. In many tumors, tumor-associated macrophages (TAMs) represent a major component of the leukocytic infiltrate [16]. High macrophage numbers have been reported in breast, ovarian, prostate, pancreatic and cervical cancers and are associated with poor prognosis [16], [17]. Some authors have characterized TAMs as macrophages expressing protumoral functions, including promotion of tumor angiogenesis, metastasis, matrix remodelling and suppression of adaptive immunity [16], [18], [19], [20]. Similar results have recently been obtained for tumor associated neutrophils [21].

Removal of macrophages or neutrophils reduced the rate of tumor progression in murine tumor models [21], [22]. Evidence suggests that TAMs are tumor-educated macrophages that appear to have defective production of reactive oxygen and nitrogen intermediates and are impaired in phagocytic activity [16]. For normal macrophages it is known that they are a primary target of virulent *Shigella flexneri* [23]. *Shigella flexneri* infection triggers caspase-1 activation leading to apotosis and processing of IL-1 and IL-18 [23].

To analyse the distribution of shigellae after i.v. application in tumor models with high numbers of macrophages (Supporting Information S1: Fig. S1), we quantified the numbers of bacteria in the extracellular space or within tumor cells, distinguishing between the macrophages and non-macrophages. Here we show that TAMs were the primary target of invasive shigellae in a 4T1 tumor xenograft - and a transgenic MMTV-HER2 - breast cancer model. Metabolically attenuated, invasive *Shigella flexneri* were almost exclusively found intracellularly, whereas a non-invasive *S. flexneri* mutant predominantly located extracellularly. Invasive *shigellae*, but not non-invasive *shigellae*, were able to activate caspase-1 and induce apoptosis in TAMs in both breast cancer models. Shigellae induced apoptosis caused a substantial depletion of TAMs, which was correlated with a pronounced and long-lasting therapeutic effect in the 4T1 breast cancer model. Finally, apoptosis induction by *Shigella* was confirmed *ex vivo* with freshly isolated human TAMs.

Taken together the data suggests that invasive bacteria capable of inducing apoptosis in macrophages might represent a novel and promising therapeutic approach for a macrophage-targeted tumor therapy.

## Materials and Methods

### Ethics Statement

All animals experiments were carried out in accordance with protocols approved by the Regierung von Unterfranken, Germany.

### Media, Chemicals and Other Reagents

Bacteria were grown on trypticase soy broth, trypticase soy agar (Becton Dickinson), LB broth, LB Agar (Sigma-Aldrich) or BHI (Becton Dickinson). Ampicillin-, chloramphenicol-, and kanamycin-resistant transformants were selected on agar containing the respective antibiotic at 100, 25, and 30 µg/ml. L-arabinose (Sigma-Aldrich) was used at 1 mM for gene expression induction. TSA containing 100 mg/l of Congo red dye was used to select Cr+ clones of *S. flexneri*. [24] Oligonucleotides (Eurofins MWG Operon), Enzymes (Fermentas) and *Taq* polymerase (Biotherm, Genecraft) were used according to manufacturers' instructions. Qiagen products were used to isolate plasmid DNA, gel-purify fragments, or purify PCR products.

### Bacterial Strains, Growth Conditions

The *S. flexneri* serotype 5a strains used in this study are the wt M90T (streptomycin (Sm) resistant] and its noninvasive variant BS176 (lacking the virulence plasmid pWR100) [25], [26]. Strains containing pKD46 and pCP20 were incubated at 30°C unless otherwise noted.

### Generation of *Shigella* aroA-Mutants

Linear DNA containing antibiotic resistance genes were prepared from the plasmids pKD3 according to the method described by Datsenko and Wanner [27]. Briefly, a PCR-product was generated by using primers AroA_up (GGGGTTTTTATTTCTGTTGTAGAGAGTTGAGTTCATGGAATCGTGTAGGCTGGAGCTGCTTC) and AroA_down (GGCCGTGCATTTGGGATCAAGAATCGTCACTGGTGTATCTGCATATGAATATCCTCCTTA) with 36 nucleotide extensions that are homologous to the *AroA*-gene. As a template, the pKD3 plasmid, which carries a chloramphenicol resistance gene flanked with FRT sites, was used. The PCR-product was transformed into electrocompetent *Shigella flexneri* BS176, carrying the recombinase encoding plasmid pKD46. In positive clones, the resistance cassette was eliminated using the flipase encoding helper plasmid pCP20 and finally the temperature sensitive helper plasmid was eliminated.

The resulting strain *Shigella flexneri* BS176ΔaroA (termed BS176*ΔaroA*) was transformed with the virulence plasmid pWR100 isolated from *S. flexneri* M90T and pCP20 as helper plasmid. After selection and elimination of the helper plasmid the mutant *S. flexneri* BS176*ΔaroA* pWR100 was obtained. The mutant carries the main feature of the virulent strain M90T and therefore was termed M90T*ΔaroA* (M90T*Δ*).

### Determination of CFU and Number of Infected Cells

CFUs were determined by plating serial dilutions in PBS containing 0.1% Triton-X (Roth) and plating on LB agar plates for *shigellae* strains. Colonies were counted after incubation overnight. For determination of the number of infected cells, serial dilutions were made in PBS, mixed with 5ml of 50°C heated SeaPlaque Agarose (Biozym Scientific) and dropped on LB agar plates. The number of bacterial colonies was counted after overnight incubation. Every colony marks an infected eukaryotic cell.

### HeLa Cell Invasion Assays and Survival Assay

Invasion and survival in HeLa (American Type Culture Collection, ATCC; CCL-2) cells was assessed using a modified gentamicin protection assay as previously described [28]. Briefly, six-well plates were seeded with 2 ml of HeLa cell suspension adjusted to 2×10^5^ cells/ml and incubated overnight to reach 90% confluency. Log-phase (OD_600_: 0.6–0.8) cultures of bacteria were added at an estimated MOI of 100 and plates were incubated at 37°C for 1 h. Subsequently plates were washed three times with PBS and then incubated with DMEM containing gentamicin (100 µg/ml) for 1 h at 37°C in 5% CO_2_. HeLa cells were lysed in a 0.1% Triton X-100 solution for 10 min at different time points and CFU was determined.

### Cell to Cell Spreading Assay

To determine cell to cell spread with a growth attenuated strain a new assay was employed. HeLa cells (7×10^5^) grown in 6-well tissue culture plates were infected at a MOI of 500 for 1 h and then washed twice with PBS. Infected cells were irradiated at 20 Gray. Subsequently, a monolayer of uninfected HeLa cells grown in 6 well plates was incubated with the irradiated *Shigella*-infected HeLa cells in a ratio of 70∶1 for 2 h, 8 h and 12 h. After 1 h incubation with 100 µg/ml gentamicin, the concentration of gentamicin was reduced to 10 µg/ml. The number of infected cells was determined as described above.

### Analysis of Caspase-1, Caspase-3, IL-1β, IL-18 and PARP Processing by Western Blot

Up to 1×10^6^ cells were washed twice with PBS and lysed in 120 µl of 2x Laemmli buffer at 100°C. 10–30 µl of lysates were separated by SDS-PAGE and transferred onto nitrocellulose membranes (Schleicher & Schuell). After 1 h blocking in 5% skimmed milk (Applichem), the membranes were incubated with the primary antibodies (anti-caspase-1/ICE, Sigma); anti-cleaved caspase-3 antibody (NEB), anti-IL1β antibody (BioVision), anti-IL18 antibody (BioVision) anti-cleaved PARP antibody (BD Pharmingen), anti-GAPDH antibody (Chemicon international) or anti-β-actin antibody (Sigma-Aldrich) diluted in 5% skimmed milk overnight at 4°C. After washing, the blot was incubated with peroxidase-conjugated secondary antibody (GE Healthcare). Detection was performed using the ECL kit (Amersham Biosciences) and exposure on X-ray film (Kodak).

### Mice and Tumor Inoculation

Six- to eight-week-old female mice were injected subcutaneously with either 1×10^4^ 4T1 cytokeratine positive murine epithelial mammary cancer cells (ATCC, CRL-2539), 1×10^6^ B78-D14 [29] melanoma cells or 1×10^6^ P815-PSA [30] mastocytoma cells each resuspended in 50 µl PBS. Injections were done on both sides of the shaven abdomen.

Balb/c, C57/BL6, DBA-2 mice were obtained from Harlan Winkelmann GmbH, Germany. Transgenic MMTV-HER2/new FVB mice were obtained from Jackson Laboratory [31]. All animals were housed at the MSZ animal care facility and experiments were carried out in accordance with protocols approved by the Regierung von Unterfranken, Germany.

### Histological and Immunohistochemical Analysis of Tumors

Tumors grown to approximately 1.5 cm in diameter were excised aseptically. The tumors were formalin-fixed, sectioned, and stained with hematoxylin and eosin (Sigma-Aldrich).

To identify macrophages at the tumor site, tissues were fixed in 4% buffered paraformaldehyde for one day, paraffin-embedded, and processed for sectioning. Sections were immunostained using the pan-macrophage anti-F4/80 rat monoclonal antibody (Acris Antibodies) and specific reactivity was detected using a peroxidase-based detection kit (Vector Laboratories). Inflammation by immune cells was detected using an anti-CD45 antibody (BD Pharmingen) and the peroxidase-based detection kit. For identification of epithelial cells an anti-Cytokeratin antibody (DAKO) and a biotinylated anti-IgG antibody (DAKO) were used.

### Assessment of *In Vivo* Targeting and Apoptosis Induction

*Shigella* were harvested in mid-logarithmic phase, washed three times in PBS and diluted in PBS prior to injection. CFU was determined before by plating serial dilutions of infection aliquots. 4T1 tumor-bearing Balb/c mice 14 days post cell implantation or approx. 6 months old female MMTV-HER2 mice bearing spontaneous mammary carcinomas were injected with 1×10^6^ CFU of bacteria into the tail vein. Bacterial titers were confirmed by plating serial dilutions. Mice were sacrificed at different time points and organs were removed. Organs were digested in 0.001% DNAse (Sigma-Aldrich) and 2 µg/ml dispase solution (Invitrogen) for 30 min at 37°C and homogenized with 70 µm and 40 µm cell strainers. Cell numbers of every cell fraction were counted using a Bürker counting chamber. For separation of cellular populations, parts of the cells were labelled with the pan-macrophage anti-F4/80 (anti-mouse IgG, Acris Antibodies; anti-human IgG, Santa Cruz) antibody for 20 minutes at 4°C. Subsequently, labelled cells were stained with an anti-IgG antibody conjugated to magnetic beads (Miltenyi-Biotec) for 10 minutes at 4°C. Cell separation was performed with the MACS Kit (Mitenyi-Biotec) according to the manufacturers protocol. Subsequently, every cell fraction was counted using a Bürker counting chamber. The CFU, the number of infected cells, caspase-1 processing and apoptosis induction was determined for every cell fraction. To determine the fraction of intra- and extracellular bacteria, CFU of total cells was determined in the presence or absence of 300 µg/ml gentamicin. For absolute values, detection limit is 10 for CFU determination and 1 for infected cells. For values relative to the cell numbers, detection limit is calculated for each experiment using the limit for absolute values divided by the highest number of cell counts for tumor cells, the macrophage or non-macrophage fraction. Detection limits for spleen cells (not shown) are usually 10 fold lower due to higher cell numbers.

### FACS Analysis

The relative amount of macrophages in tumor tissues was determined by FACS. After blocking the Fcγ receptor (anti-CD16/32, BD Pharmingen), cells were stained with FITC-anti-mouse CD11b (Miltenyi-Biotec; 10 minutes at 4°C), phycoerythrin (PE)-anti-mouse Gr-1 (Miltenyi-Biotec; 10 minutes at 4°C) or PE-anti-mouse F4/80 (Acris Antibodies; 30 minutes at 4°C). FITC or PE coupled IgG_2B_ antibody (RD Systems,) were used as isotype control. Total cell numbers were assessed in a FSC/SSC live gate. Flow cytometric analysis was performed on a FACSCalibur (BD Immunocytometry Systems) according to the manufacturer's instructions.

### Efficacy Studies

Therapeutic efficacy of *Shigella* infection on tumor growth was explored in 28 six- to eight-week-old female Balb/c mice bearing 4T1 tumors. Tumor growth was determined every two days with a caliper. At day 14 post cell implantation, mean tumor volume had reached 170 mm^3^ and 24 mice were randomized into three groups. A total amount of 1×10^6^ M90T*ΔaroA*, BS176*ΔaroA* or 100 µl PBS were injected into the lateral tail vein of the tumor-bearing mice. Tumor size was monitored every second day. Tumor volume was calculated with the formula V = π/6*a*b^2^ (a>b). 31 days after tumor cell implantation the naϊve and the *BS76ΔaroA* group (tumor volume of approx. 4000 mm^3^) and two M90T*ΔaroA* mice (macroscopic comparison of tumors, data not shown) were sacrificed. On day 62 post tumor cell implantation M90T*ΔaroA-infected* mice were sacrified to determine CFU in tumor, liver, spleen and for FACS and histological analysis.

### Statistical Analysis

Statistical analysis was performed using Graph Pad Prism 4 (GraphPad Software). Data were compared and analyzed for statistical significance by using a two-tailed Student's t-test whereas p<0.05 was considered as significant.

## Results

### Generation and *In Vitro* Characterization of Attenuated Shigella Strains

For infection, *S. flexneri* serotype *5a* strains M90T and BS176 were used. BS176 is a variant of M90T lacking the virulence plasmid [32]. An auxotrophic and attenuated strain for animal studies was constructed, by chromosomal deletion of the *aroA*-gene locus. Isogenic virulent and avirulent strains were generated by attenuating *Shigella flexneri* strain BS176 to yield BS176*ΔaroA* followed by transfer of the virulence plasmid pWR100 [25]. The resulting mutant was called M90T*ΔaroA.*

**Figure 1:**
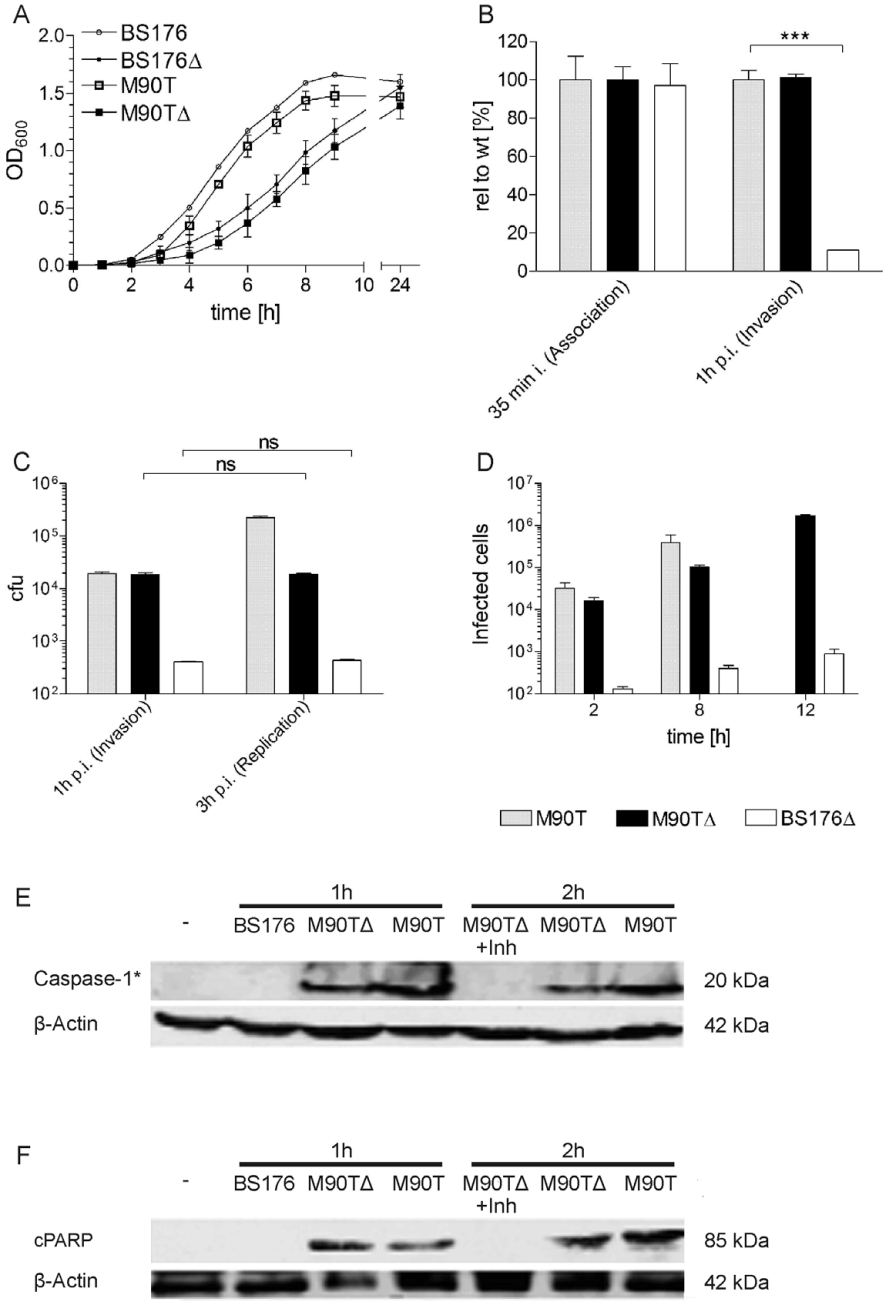
*In vitro* characterization of aroA mutants. (A) Determination of extracellular growth rate in LB medium. (B) Invasion assay (gentamicin protection assay). CFUs are shown relative to the wt M90T. (C) Intracellular replication rate in HeLa cells. (D) Cell to cell spread in HeLa cells assessed by L-Top agar assay. Western blot analysis for caspase-1 activation (caspase-1*) (E) and apoptosis induction was performed in J774A.1 Macrophages (PARP cleavage - cPARP) (F). Bars represent means +/- SD of three different experiments, *** P<0.001. BS176: *Shigella flexneri* BS176; BS176Δ: *Shigella flexneri* BS176*ΔaroA*; M90T: *Shigella flexneri* M90T; M90TΔ: *Shigella flexneri M90TΔaroA*.

The strains were characterized with respect to extracellular and intracellular growth, adhesion, invasion and cell-to-cell spread *in vitro* (Fig. 1). The attenuated mutants had a reduced maximal growth rate in LB medium compared to the parental strains with functional *aroA*-genes (Fig. 1A). With respect to association and invasion, *S. flexneri* M90T*ΔaroA* was indistinguishable from the M90T strain (Fig. 1B). Consistent with the behaviour of other *aroA* deleted mutants the intracellular replication was reduced (Fig. 1C). To determine the cell-to-cell spread, a spreading assay was applied, which is independent of intracellular replication (see material and methods section). *S. flexneri* M90T*ΔaroA* was actively spreading as a 6- or 17-fold increase of infected cells was seen at 8 vs 12 hours post coincubation (Fig. 1D). In contrast the isogenic control BS176*ΔaroA* was only marginally active (Fig. 1D).

The capacity of *S. flexneri* M90T*ΔaroA* to induce both caspase-1 cleavage (Fig. 1E) and apoptosis (Fig. 1F) was confirmed in J774A.1 macrophages by western blot analysis (Supporting Information S1: Fig. S2). Apoptosis induction by *S. flexneri* M90T*ΔaroA* was caspase-1 dependent, as the caspase-1 specific inhibitor YVAD-CHO fully blocked caspase-1 and PARP processing (Fig. 1E).

### Infection of TAMs by Shigella *In Vivo*

In the next set of experiments, 1×10^6^ invasive *S. flexneri* M90T*ΔaroA* or non-invasive BS176*ΔaroA* derivative were applied i.v. to 4T1 xenografted Balb/c or tumor-bearing transgenic MMTV-HER2 mice. After tumor removal, single cell suspensions were prepared and treated with or without gentamicin to determine the ratio of extra and intracellular bacteria. Parts of the tumor cell suspension were separated by MACS into a TAM fraction and TAM-depleted fraction using an F4/80 antibody (Supporting Information S1: Fig. S3,4,5). Supporting Information S1: Fig. S3,4 also depicts the phenotypic characterization of the TAMs. 83% of CD11b positive cells were F4/80 positive (Supporting Information S1: Fig. S3). The F4/80 positive population is DC-M negative, MHC class II low and Gr-1 negative (Supporting Information S1: Fig. S4). The number of infected cells was determined by plating in L-Top agar.

**Figure 2:**
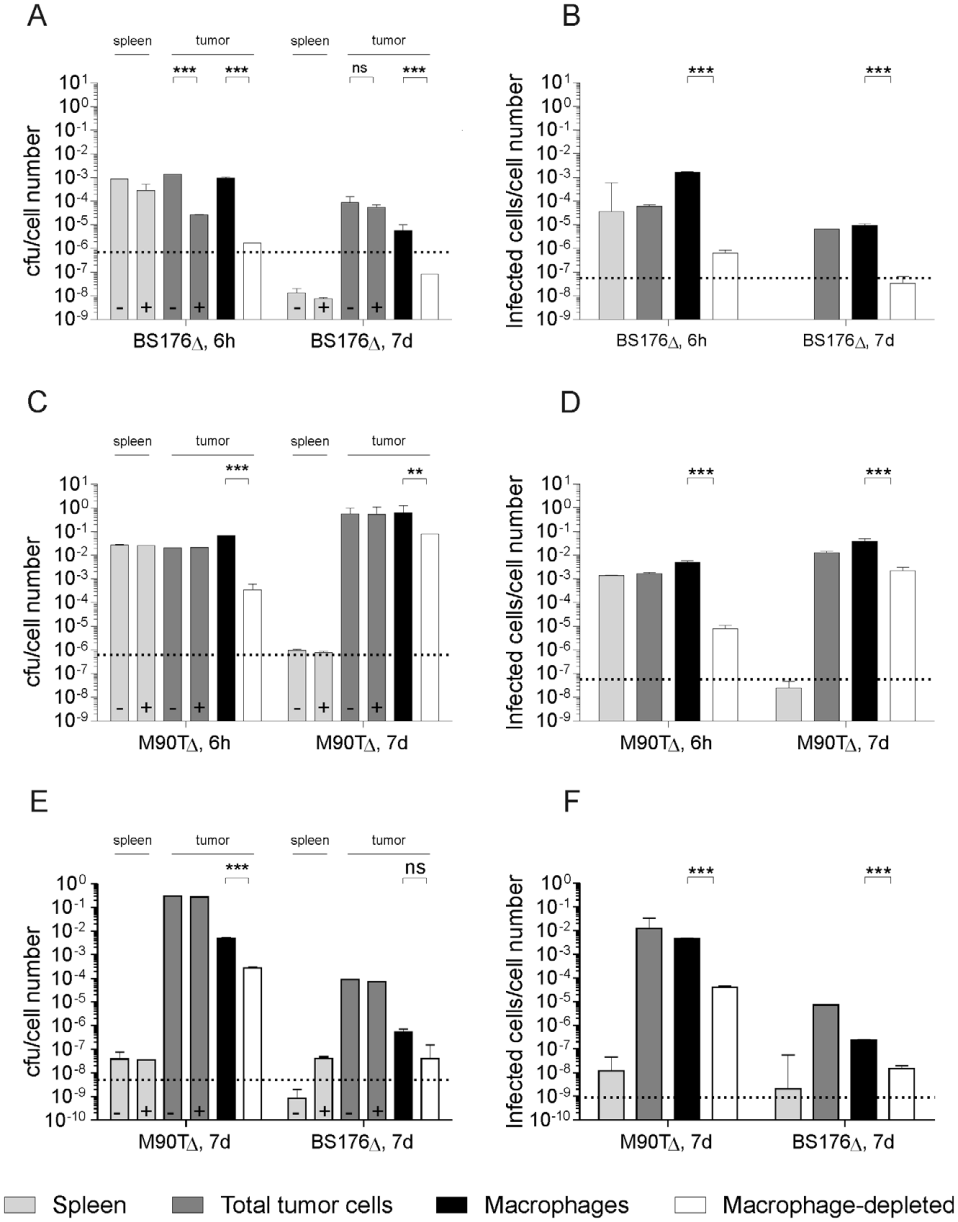
*Shigella flexneri* M90T*ΔaroA* predominantly targeted TAMs *in vivo*. Determination of CFU/cell number (A, C, E) and infected cells/cell number (B, D, F) of separated tumor cells and spleen cells 6 h and 7d after i.v. infection with 1×10^6^ bacteria of tumor-bearing 4T1 (A–D) and transgenic MMTV-HER2/neu mice (E, F). Corresponding raw data of CFU and infected cell counts are shown in Supporting Information S1: Fig. S6 and Supporting Information S1: Fig. S7. + indicates gentamicin treatment. Dashed lines indicate detection limits. All results shown are mean ± SD of values from three mice per experiment; ** P<0.01, *** P<0.001. BS176Δ: *Shigella flexneri* BS176*ΔaroA*; M90TΔ: *Shigella flexneri* M90TΔ *aroA*

Similar to observations with other bacterial strains, both invasive and non-invasive *Shigella* accumulated and persisted in the tumor after i.v. infection, whereas they were rapidly eliminated in the spleen (Fig. 2*A, C*). However, both strains showed marked differences with respect to their intra-tumoral distribution. In the 4T1 tumor model, gentamicin treatment revealed that 6 h after infection the invasive *S. flexneri* M90T*ΔaroA* almost exclusively located intracellularly, whereas only 2% of the non-invasive derivative were located intracellularly (Fig. 2*A*). Seven days after application invasive *Shigella* reached total CFU levels of up to 6×10^6^ per tumor. Unexpectedly, the invasive strain was predominantly located in TAMs (>10 fold enrichment, Fig. 2C) even though M90T*ΔaroA* readily infected epithelial 4T1 cells in culture (Supporting Information S1: Fig. S11). In case of the non-invasive BS176*ΔaroA*, only a small amount of bacteria were found that were restricted to macrophages 6 h p.i. (*Fig. 2A*), in line with previous reports [5]. Seven days after infection, non-invasive shigellae remained restricted to TAMs and were at the limit of detection (Fig. 2A). In contrast the ratio of invasive bacteria in TAMs vs non-TAMs was decreased at seven days p.i. presumably due to cell to cell spreading as observed in cell culture (Fig. 1E,F).

The predominant localization in TAMs was not due to higher CFU counts per cell compared to non-macrophages, as the infectious center assay led to the same results (Fig. 2B, D). The selective targeting of invasive *shigellae* to TAMs was also observed in tumor bearing MMTV-HER2 transgenic mice (Fig. 2E, F).

Taken together, non-invasive BS176*ΔaroA* shigellae were mainly found extracellularly despite the presence of large numbers of macrophages in two different breast cancer models, whereas invasive M90T*ΔaroA* shigellae almost exclusively targeted TAMs.

### Apoptosis Induction in TAMs

**Figure 3:**
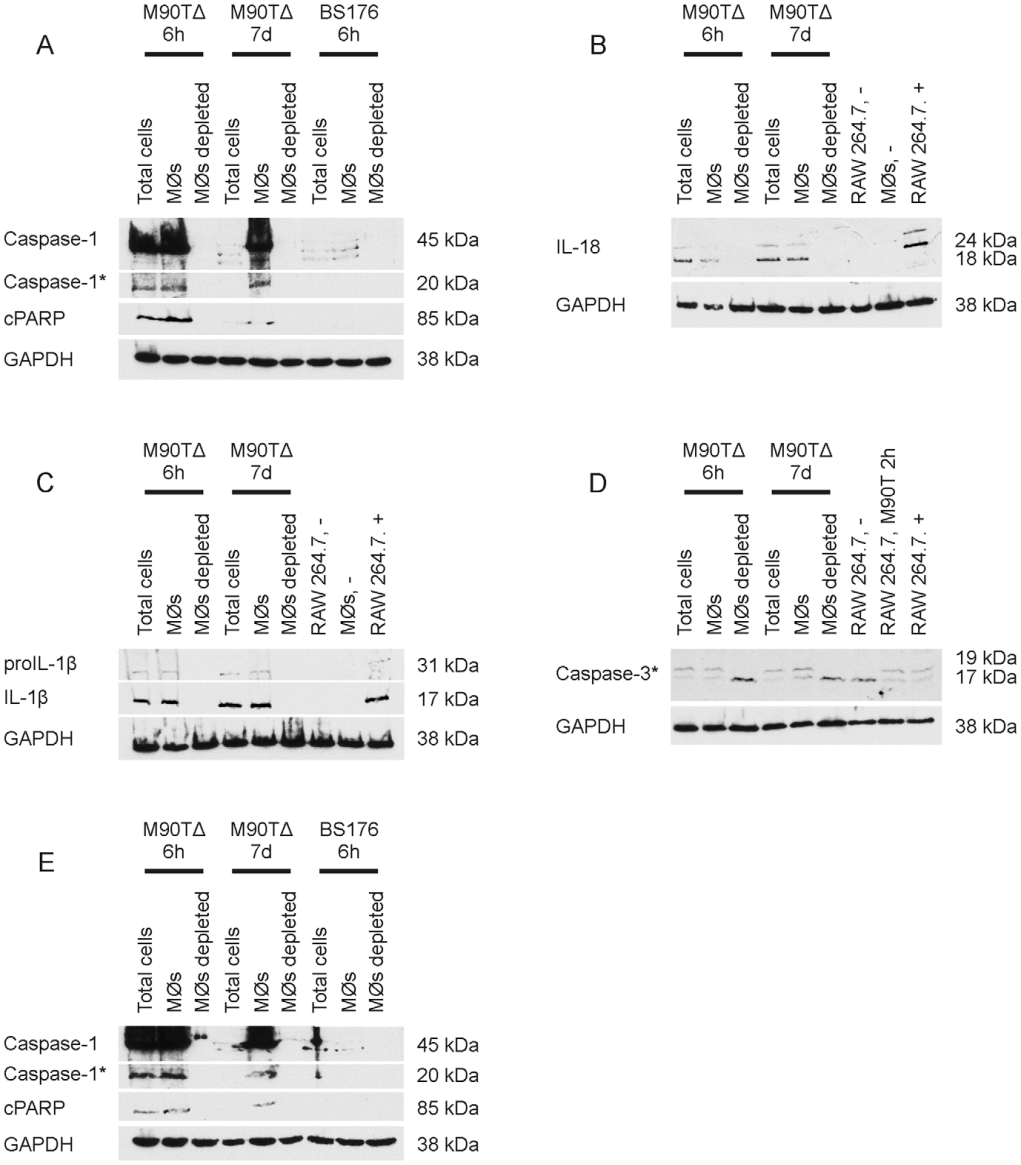
Infection of tumor-bearing mice with *Shigella flexneri* M90T*ΔaroA*, but not *S. flexneri* BS176*ΔaroA* induced consistent caspase-1 processing and apoptosis in TAMs in vivo. Western Blot analysis for caspase-1 (A, E), IL-1β (C), IL-18 (B), caspase-3 (D) processing and PARP cleavage (A,E) in Balb/c (A–D) and transgenic MMTV-HER2 (E) mice. BS176Δ: *Shigella flexneri* BS176*ΔaroA*; M90TΔ: *Shigella flexneri* M90TΔ*aroA*

To examine whether the apotosis induction observed in the established macrophage cell line J774A.1 in culture also occurred in TAMs in vivo, tumor bearing mice were infected. After infection with invasive M90T*δaroA* shigellae, caspase-1 activation (Fig. 3*A*) was detectable in total cells and macrophage fractions of tumors taken 6 hours after infection. At seven days p.i. caspase-1 activation was exclusively seen in the macrophage fraction. Caspase-1 activation at either timepoint was invariantly associated with IL-18 and IL-1β processing (Fig. 3*B,C*) in addition to processing of the effector caspase-3 and PARP cleavage (Fig. 3A,D), a hallmark of apoptosis. In contrast the non-invasive BS176*ΔaroA* did not induce apoptosis. Apoptosis induction was only observed in cellular fractions, where *shigellae* were located intracellularly and that contained TAMs. Similar data were obtained for transgenic MMTV-HER2 mice (Fig. 3*E*). In both mouse models TUNEL staining in tumor tissue confirmed caspase-3 processing and PARP cleavage data (Supporting Information S1: Fig. S8).

**Figure 4:**
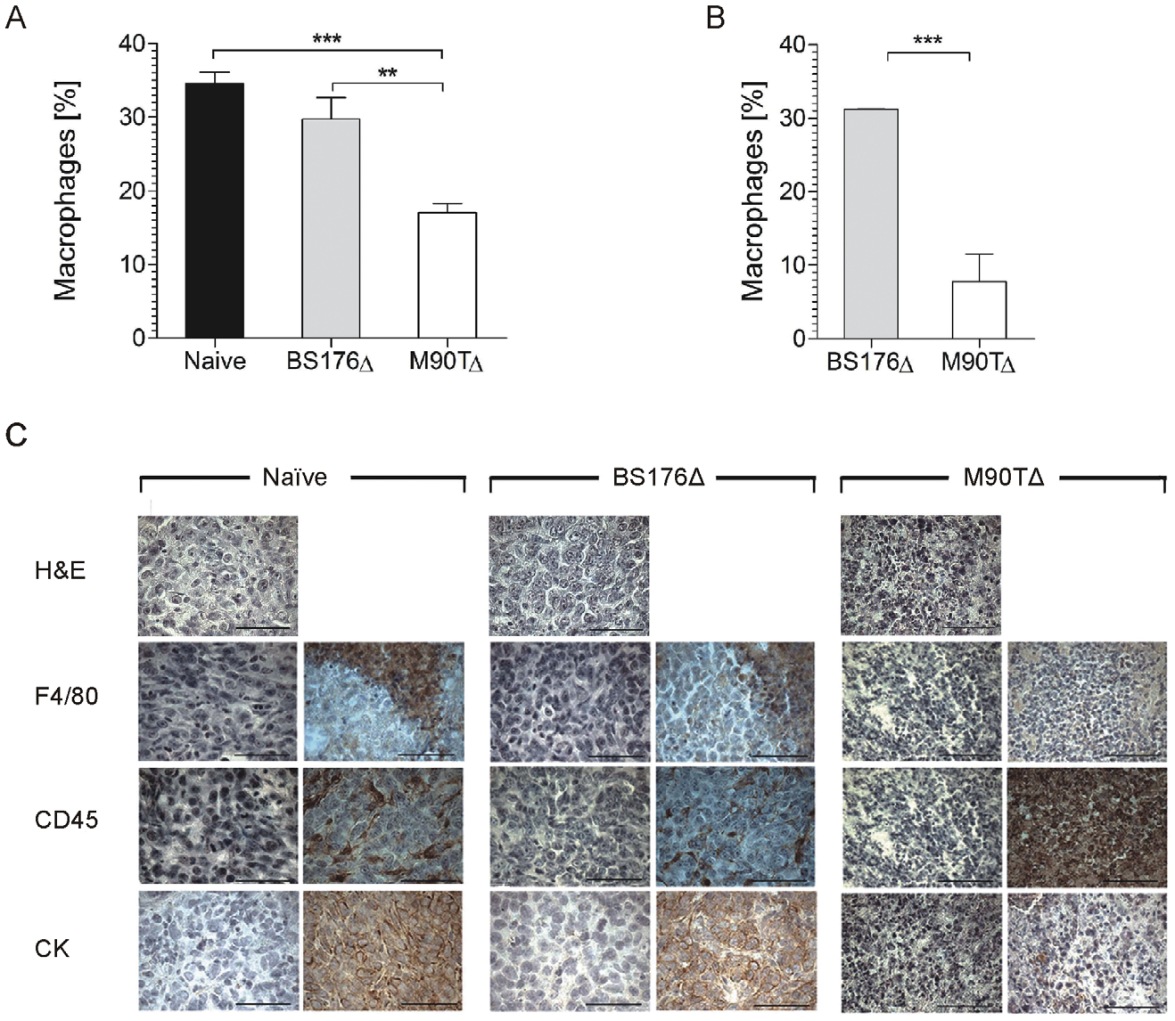
Infection with *Shigella flexneri* M90T*ΔaroA* results in a decrease in TAMs number. 7d after IV infection of tumor bearing Balb/c (A) and MMTV-HER2 FVB mice (B) tumors were analysed for F4/80 positive cells by FACS to determine the relative amount of TAMs. Bars represent means +/− SD of four tumors analyzed per group, ** P<0.01, *** P<0.001. (C) Histology of tumor tissue from non-infected, BS176Δ- or M90TΔ- infected mice. In each case left panels represent antibody controls. Scale bars represent 50 µm. BS176Δ: *Shigella flexneri* BS176*ΔaroA*; M90TΔ: *Shigella flexneri* M90TΔ*aroA*; CK: Cytokeratin.

Next we investigated whether apoptosis induction in vivo was associated with depletion of macrophages. Figure 4 shows that M90T*ΔaroA*, but not the avirulent shigellae reduced the relative content of TAMs in tumors from xenografted Balb/c mice by at least 50% seven days p.i. (Fig. 4*A*). In transgenic mice a 74% reduction was achieved by M90T*ΔaroA* but not control shigellae infection at the seven day timepoint (Fig. 4B). This result cannot be explained by differences in tumor sizes between animals (Supporting Information S1: Fig. S9) and therefore strongly suggests that infection with invasive bacteria induced the depletion of TAMs. In addition, the reduction of macrophages is not observed in spleens of infected animals suggesting that the reduction is confined to tumor tissues (data not shown).

Histological examination of non-infected (Fig. 4C left panels), BS176*ΔaroA* (Fig. 4*C*, middle panels) and M90T*ΔaroA* (Fig. 4C right panels) infected mice by anti-F4/80 staining confirmed the substantial reduction of macrophages. Additionally the IHC experiments revealed massive infiltration of inflammatory cells (anti-CD45 staining) in 4T1-tumors upon infection with invasive but not non-invasive Shigella (Fig. 4C). Furthermore, cytokeratin staining of tumors derived from M90T*ΔaroA*-infected mice, which identifies pockets of 4T1 tumor cells showed reduced content of cytokeratin positive cells. This result indicates that the depletion of macrophages is associated with a reduction of tumor cells as previously observed by other approaches targeting macrophages [19], [33], [34]. In the non-infected and BS176*ΔaroA*-infected tissue normal tumor structure was observed.

### Tumor Regression by Depletion of TAMs

**Figure 5:**
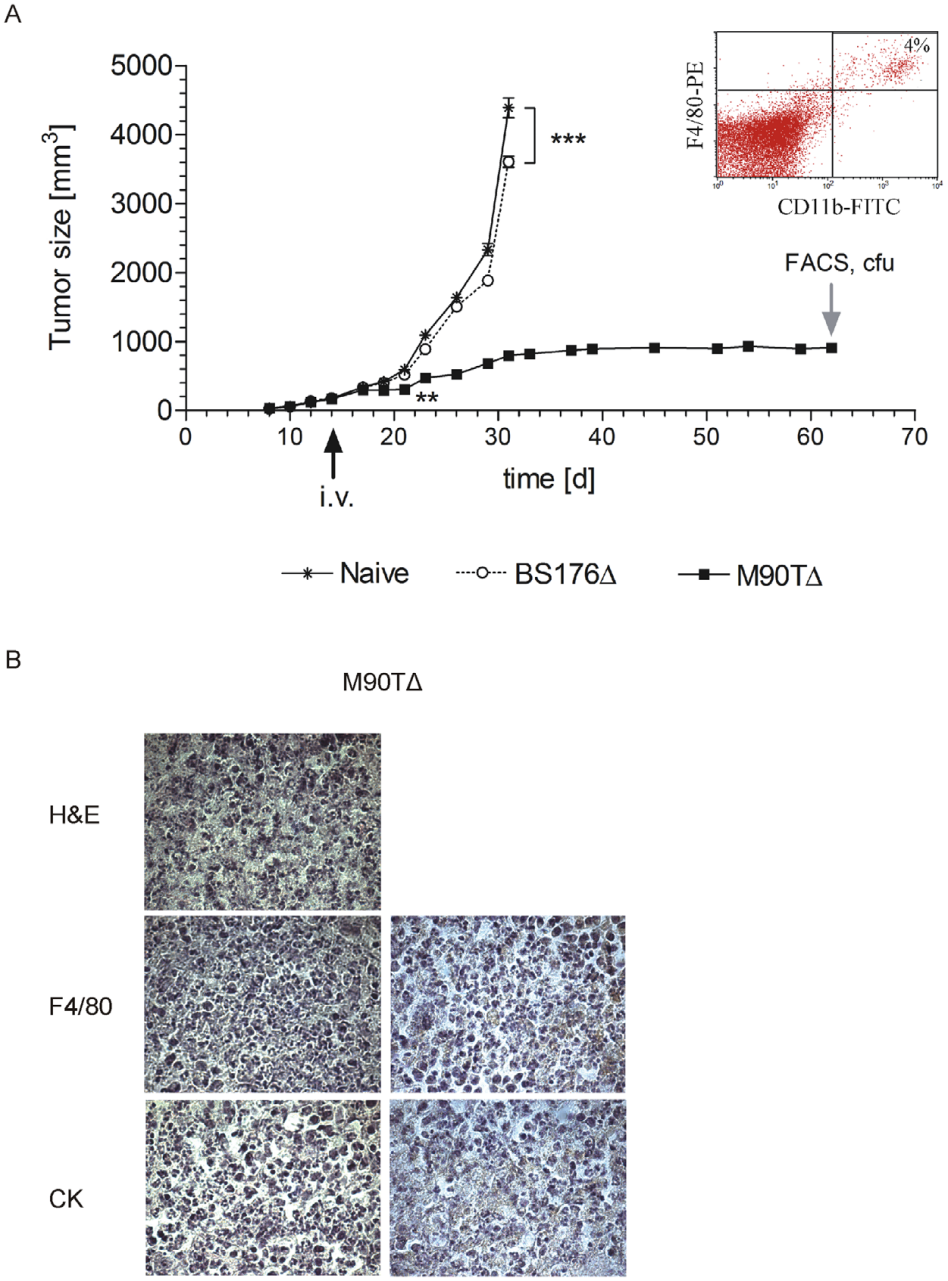
M90T*Δ*, but not BS176*Δ*, completely blocked tumor growth after i.v. infection of 4T1 tumor-bearing mice. (A) 14 days after tumor cell inoculation 1×10^6^ bacteria were applied i.v. to 8 mice per group. Non-infected and BS176Δ infected mice were sacrificed 31 days after tumor cell inoculation. At day 62, M90TΔ mice were killed and CFU and macrophage numbers were determined. ** P<0.01, *** P<0.001. (B) Histology of tumor tissue from M90TΔ-infected mice. Left panels represent antibody controls. Scale bars represent 50 µm. BS176Δ; *Shigella flexneri* BS176*ΔaroA*; M90TΔ; *Shigella flexneri* M90TΔ*aroA*;CK: Cytokeratin.

To investigate whether this substantial reduction in macrophage numbers and marked inflammation induced by *S. flexneri* M90T*ΔaroA* is associated with a therapeutic effect, bacteria were applied to tumor-bearing Balb/c mice and tumor growth was assessed (Fig. 5*A*). Infection with *S. flexneri* BS176*ΔaroA* resulted in a small, albeit significant reduction of tumor growth in comparison to the control animals. In contrast, a single i.v. infection with *S. flexneri* M90T*ΔaroA* resulted in complete removal of tumor tissue (Fig. 5). 48 days p.i. the tumor showed very low macrophage (<4%) numbers and bacteria were not detectable (Fig. 5*A*). Histological examinations of the tumor tissue 68 days after xenografting showed persistent reduction of macrophage numbers (anti-F4/80 staining) and complete depletion of 4T1 tumor cells (anti-cytokeratin staining) (Fig. 5*B*).

## Discussion

In this work we have demonstrated that invasive *S. flexneri* preferentially target TAMs after i.v. infection in two different breast cancer models. The growth-attenuated virulent *mutant* M90T*ΔaroA*, but not the non-invasive mutant BS176*ΔaroA*, was exclusively located intracellularly at all time points examined. *Mutant* M90T*ΔaroA*, but not BS176*ΔaroA*, induced caspase-1 processing and apoptosis in TAMs leading to a long-lasting reduction of TAMs. The reduction of TAMs was accompanied by a complete tumor regression in a 4T1 breast cancer model and removal of transformed cells in the residual stroma. In contrast to bacterial tumor therapies that use extracellular bacteria, targeting TAMs with invasive shigellae was effective despite relatively low numbers of bacteria within the tumor. Finally, for a different gynaecological cancer type of human origin, human ovarian carcinoma, we could show *ex vivo* that infection of TAMs from ascites with *S. flexneri* M90T*ΔaroA* induced caspase-1 activation and PARP cleavage (Supporting Information S1: Fig. S10).

Experimental tumor therapy by infection with extracellular bacteria has a long history [3]. Recent observations with *E.coli* strains suggested that these bacteria were able to induce tumor-targeted inflammatory responses but were ineffective in killing tumor cells [5]. The non-invasive strain BS176*ΔaroA* showed a similar behavior, including a marginal, albeit significant, therapeutic efficacy. Bacterial tumor therapy is commonly thought to be based on the induction of an inflammatory response at the site of the tumor as a result of the accumulation of extracellular bacteria at this immunologically privileged site [4], [11], [35]. Whereas these bacteria remain extracellular at all times *S. flexneri* M90T*ΔaroA* actively invades cells. This data confirm previous reports which have described phagocytosis defects in TAMs [16], [19].

In our experiments, the profound reduction of TAMs was associated with complete tumor regression [36]. The simplest interpretation of these data would be that TAMs are required to support tumor growth. However as we observed a massive infiltration of inflammatory cells simultaneous with bacterial infection and TAM depletion inflammation might also contribute to tumor regression. The extracellular BS176*ΔaroA* mutant did not induce caspase-1 processing and thus did neither induce IL-1β and IL-18 activation nor deplete TAMs. Therefore it is likely that for the induction of the inflammatory response by M90T*ΔaroA* targeting of macrophages is also a necessary factor. However the low dose bacteria found under these conditions makes it difficult to come up with a firm conclusion in this respect. As neutrophils have been described to be potential mediators of inflammation induced tumor regression [21] it may be interesting in the future to determine whether M90T*ΔaroA* can induce neutrophil infiltration in the presence or absence of TAMs.

Targeting TAMs as a strategy for bacterial tumor therapy may have the advantage that a stable cell population is attacked as opposed to the phenotypically unstable tumor cell population, that may quickly give rise to resistant cells. On the other hand macrophages found in tumors may potentially stimulate or inhibit tumor growth. It will therefore be important in the future to develop markers that differentiate between TAM populations and allow identification of tumors where TAM removal may be beneficial [37], [38]. Although at first sight *Shigella* would seem to be an unlikely candidate as a therapeutic agent based on its pathogenicity, the fact that attenuation was readily achieved and that a small number of bacteria at the tumor site was sufficient to induce a dramatic anti-tumor effect suggests that further investigation is warranted. At least in our experiments we did not observe signs of overt toxicity, albeit mice cannot be considered as a suitable model to assess toxicity of shigellae.

In summary, we suggest that targeting TAMs using attenuated *S. flexneri* is a promising option for future tumor therapy. Further studies are required with respect to the safety of the *S. flexneri* mutant and the efficacy of tumor targeting in humans. Furthermore, other intracellular bacteria like *Salmonella enterica* or *Listeria monocytogenes* might be suitable for a macrophage targeted bacterial tumor therapy.

## Supporting Information

Supporting Information S1 (15.15 MB DOC)
