# Supplementary material for: Shigella Mediated Depletion of Macrophages in a Murine Breast Cancer Model Is Associated with Tumor Regression
Source: PLoS One. 2010 Mar 8;5(3):e9572. doi: 10.1371/journal.pone.0009572 (PMC2833200; doi:10.1371/journal.pone.0009572)
Supplement: Supporting Information S1 — (15.15 MB DOC) [file pone.0009572.s001.doc]

| Supporting information Fig. S 1:  Substantial amounts of TAMs are detectable in different mouse tumor models. |
| --- |
| 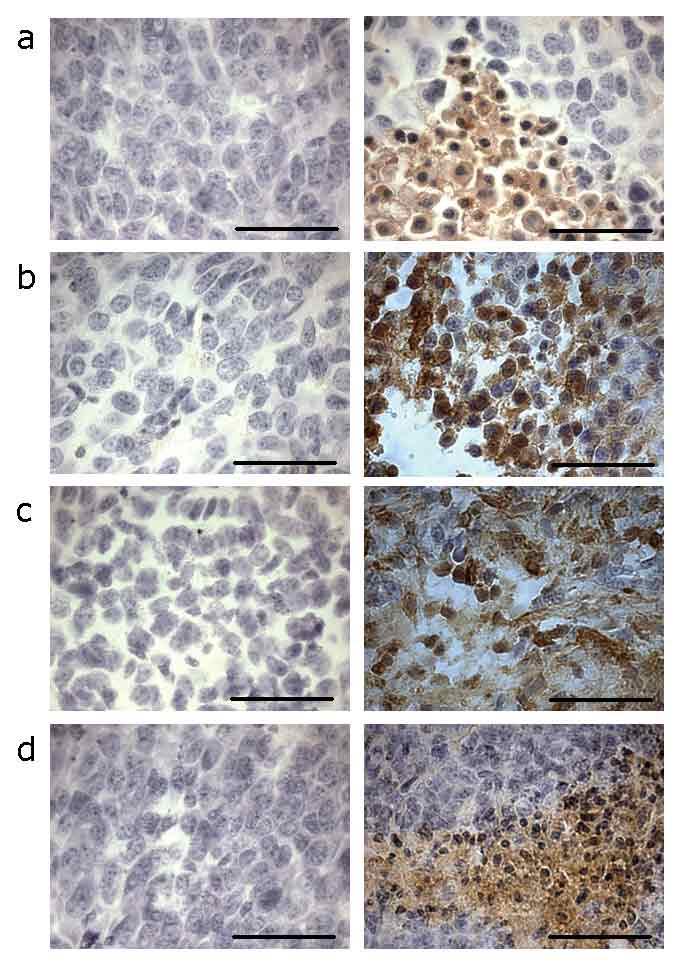 |

Tumors with diameters of 1 – 1.5 cm were isolated from mice injected s.c. with 1 x 10^6^ B78-D14 cells (a), 1 x 10^4^ 4T1 cells (b) and 1 x 10^6^ P815-PSA cells (c). In addition, spontaneous breast tumors from transgenic MMTV-HER2/new FVB were isolated (d). Tumor tissue was fixed and embedded in paraffin. Tumor sections were immunostained with a biotinylated anti-F4/80 monoclonal antibody and subsequently nuclei were counter-stained with haematoxylin (right). Staining with avidin-horseradish peroxidase without F4/80 antibody was performed as a control (left). Scale bars represent 50 µm.

| Supporting information Fig. S 2:  Caspase-1 is exclusively expressed by macrophages. |
| --- |
| 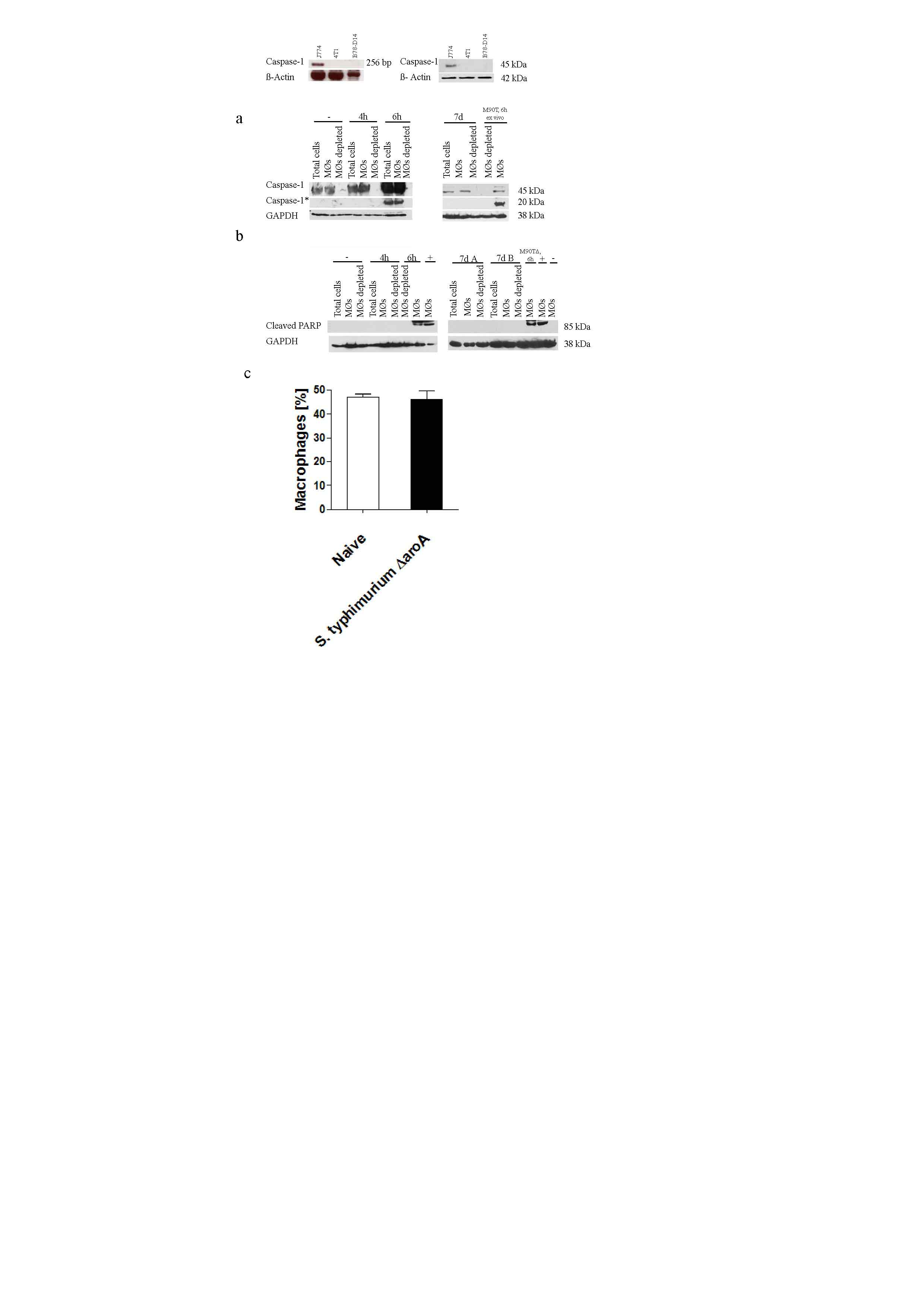 |
| Analysis of caspase-1 expression by RT-PCR (left) and Western Blot (right).  For RT-PCR RNA of up to 1 x 10^7^ cells of every cell line (B78-D14, 4T1 and J774A.1), was isolated by the protocol of RNeasy® Mini Kit (Qiagen). cDNA synthesis was performed using First Strand® cDNA Synthesis Kit (Fermentas) after manufacturers instructions. Subsequently PCR was performed with *Taq* Polymerase (Biotherm, Genecraft). To determine caspase-1 expression the primers Casp1RT_left 5`-TGCCCTCATTATCTGCAAC-3` and Casp1RT_right 5`-GGTCCCACATATTCCCTCCT-3` were used. The *ß-actin*-gene was used as control (Primer: actin s1: 5`-GTCGTACCACAGGCATTGTGATGG-3` and actin as 5`-GCAATGCCTGGGT ACATGGTGG-3`). |

| Supporting information Fig. S 3:  Experimental schedule of cell separation. |
| --- |
| 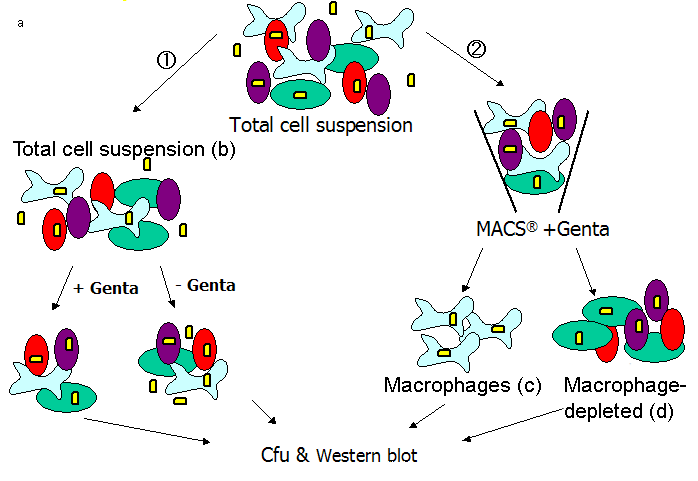  a  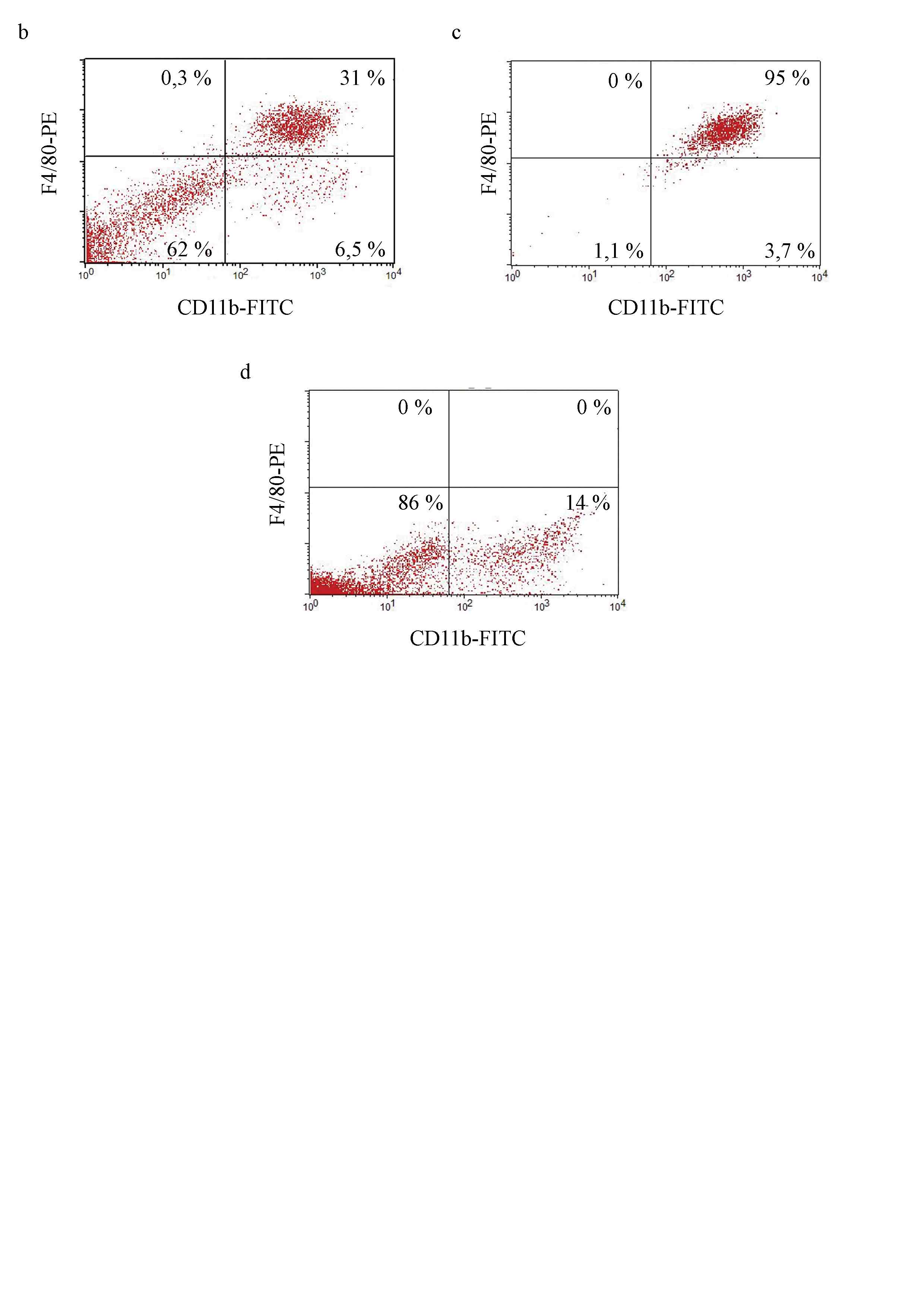 |
| (a) After tumor removal and separation of the total tumor cells by 0.001 % DNAse and 2 µg/ml dispase treatment for 30 min at 37 °C, part of the total tumor cell suspension ➀ was treated with or without 300 µg/ml gentamicin for 1 h. Afterwards, cells were analysed for CFU and caspase-1 activation. The fraction treated with gentamicin consists mainly of intracellular bacteria, whereas the untreated fraction contains extra- and intracellular bacteria. The second part of total tumor cells ➁ (b) was labeled with an anti-F4/80 (IgG) antibody. Then a bead-conjugated secondary anti-IgG antibody was added. Separation was performed using MACS columns in magnetic fields and resulted in two cell fractions: the macrophage enriched (referred to as macrophage fraction) (c) and the macrophage-depleted fraction (d). The purity of the macrophage fraction was between 95 and 99 % (n = 7) as assessed by staining for F4/80 and CD11b (see also Fig. S6). Throughout the procedure, cells were treated with 300 µg/ml gentamicin to prevent re-infection of cells by free bacteria. Using these magnetically purified fractions, the CFU and caspase-1 processing was assessed. Note that the macrophage fraction (c) contained a substantially lower amount of cells compared to the macrophage-depleted (d) fraction. For CFU counts, normalization was performed after plating (CFU/cell number, infected cells/cell number), for Western Blotting, equivalent cell numbers were loaded. |

| Supporting information:Fig. S 4:  Characterization of the macrophage fraction after F4/80 MACS separation. |
| --- |
| 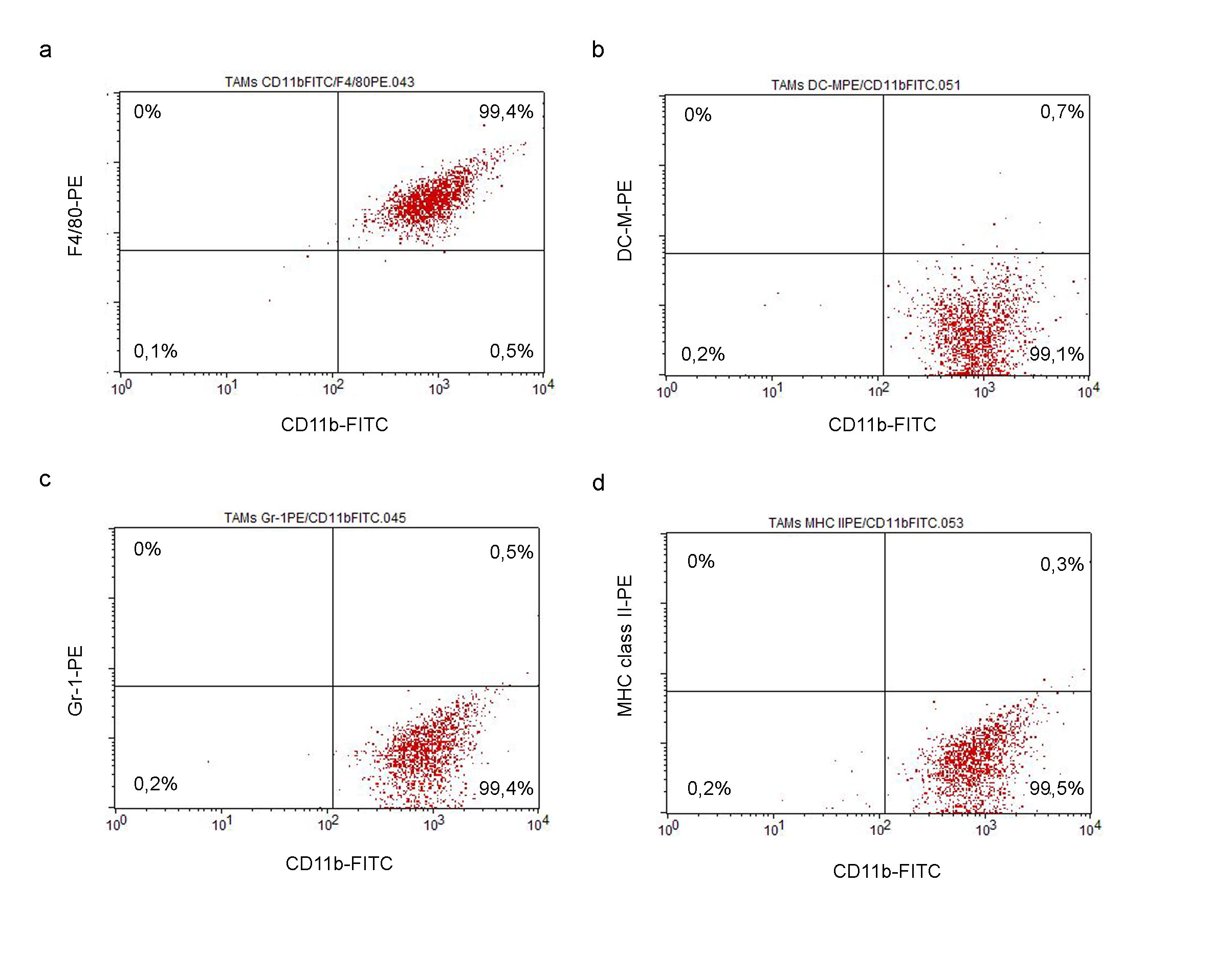 |
| For purity control, macrophage fraction (**a**) was also stained by an anti-CD11b-FITC and an anti-Dentritic Cell Marker-PE (**b**), an anti-Gr-1-PE (**c**) and an anti-MHC Class II-PE (**d**) antibody. The figure shows one representative experiment out of three. The degree of purity in the macrophage fraction was approximately 99 %. |

| Supporting information Fig. S 5:  Light microscopic analysis (x100) of the three cell fractions after cell separation. |
| --- |
| 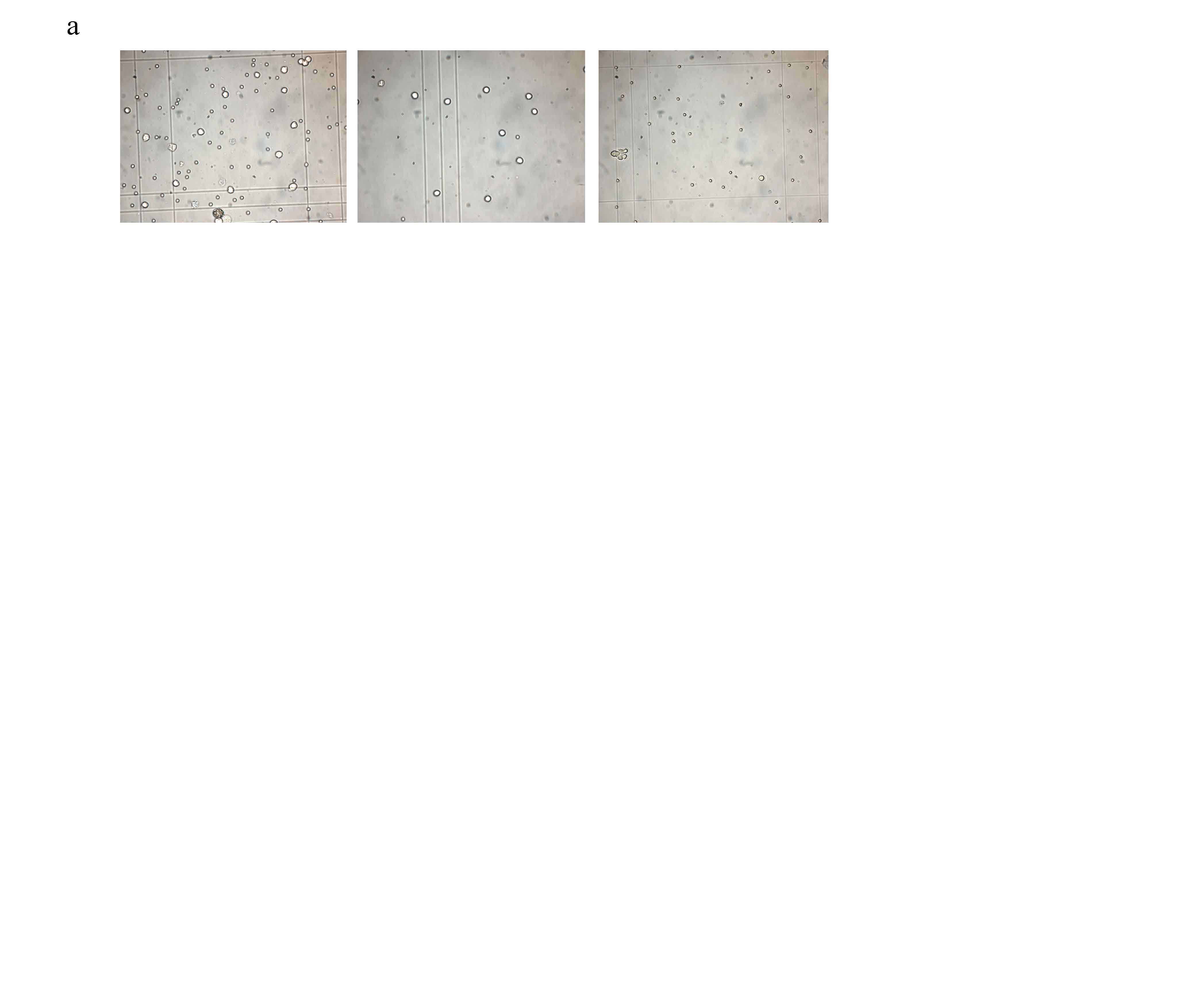 |
| To evaluate the efficacy of the cell separation which could influence the results with respect to the number of infected cells, light microscopic analysis of the three different cell fractions was performed. The left panel shows the total tumor cell fraction. In the middle the separated macrophages are depicted. The left panel represents the macrophage-depleted fraction. The cells of all fractions mainly appear as single cells, which is consistent with the FSC – SSC plots of the FACS analyses (not shown). |

| Supporting information Fig. S 6:  *In vivo* infection of tumor-bearing Balb/c mice with *S. flexneri BS176ΔaroA* (BS176Δ) and *S. flexneri M90TΔaroA* (M90TΔ).  Determination of CFU (a, c) and the number of infected cells (b, d) after 6 h and 7 d. Tumor-bearing Balb/c mice (n = 4) were infected with 1 x 10^6^ *S. flexneri BS176ΔaroA* (BS176Δ) and *S. flexneri M90TΔaroA* (M90TΔ) i.v.. At different time points *p.i.* spleens and tumors were removed, cells were separated and treated with gentamicin (+) or not (-). Total CFU was determined by plating serial dilutions of lysed cells and the number of infected cells was determined by plating serial dilutions of intact cells in L-Top agar. |
| --- |
| 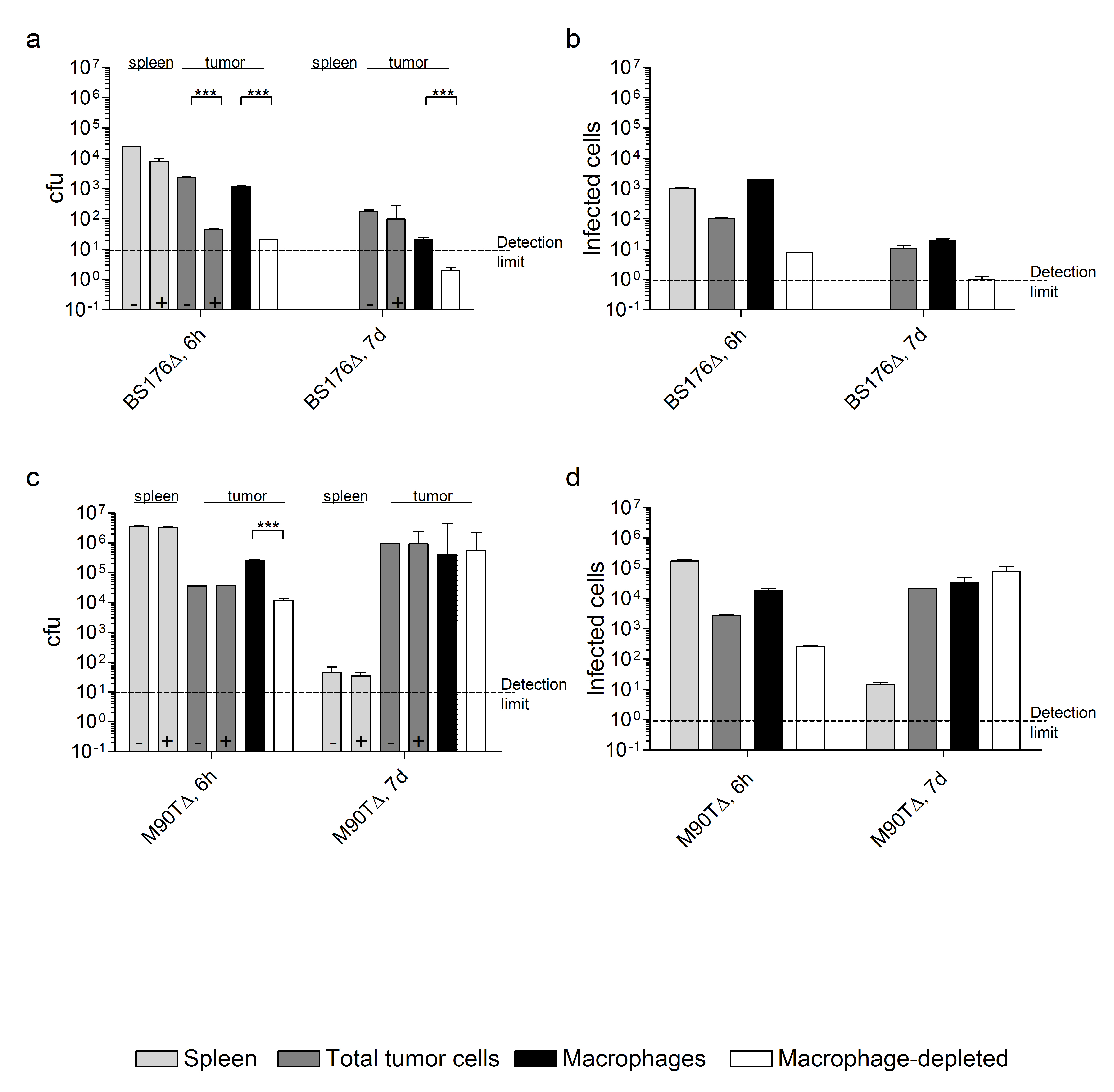 |

*Shigella flexneri M90TΔaroA* (M90TΔ) predominantly targets TAMs *in vivo*. All results shown are mean ± SD (n = 4); *** P < 0.001, Student`s t-test.

| Supporting information Fig. S 7:  Also in transgenic mice bearing spontaneous breast adenocarcinomas, *S. flexneri M90TΔaroA* predominantly localizes to macrophages in the tumor.  Total cfu (a) and infected cells (b) values determined by L-Top agar assay after 7 d. MMTV-HER2/new FVB mice (n = 4) were infected with 1 x 10^6^ *S. flexneri M90TΔaroA* and *BS176ΔaroA*. 7 d *p.i.,* spleens and tumors were removed and cells were separated. Total CFU was determined by plating serial dilutions of lysed cells and the number of infected cells was determined by plating serial dilutions of intact cells in L-Top agar. *S*. *flexneri M90TΔaroA* infects predominantly the TAMs 7 d *p.i*. |
| --- |
| 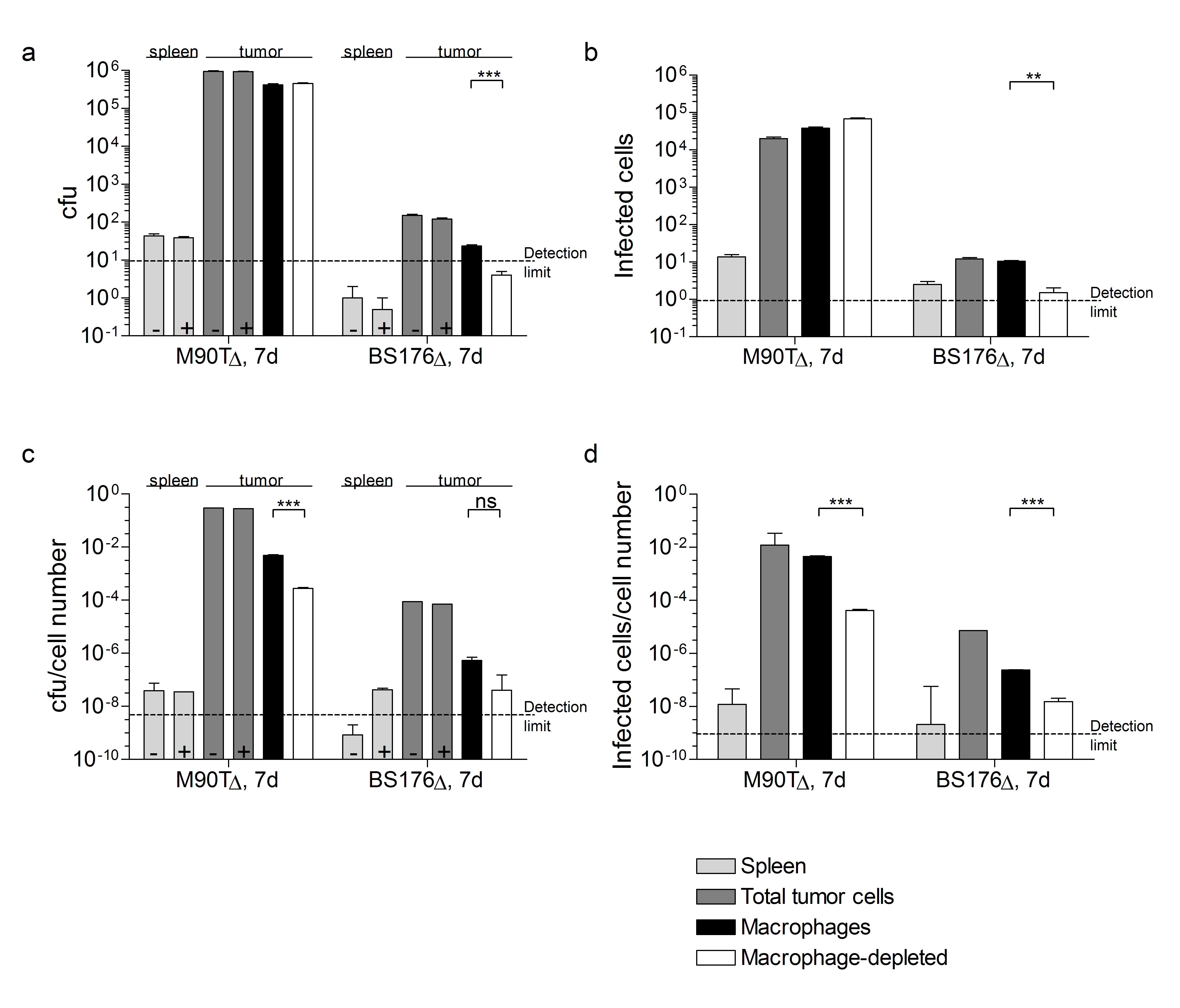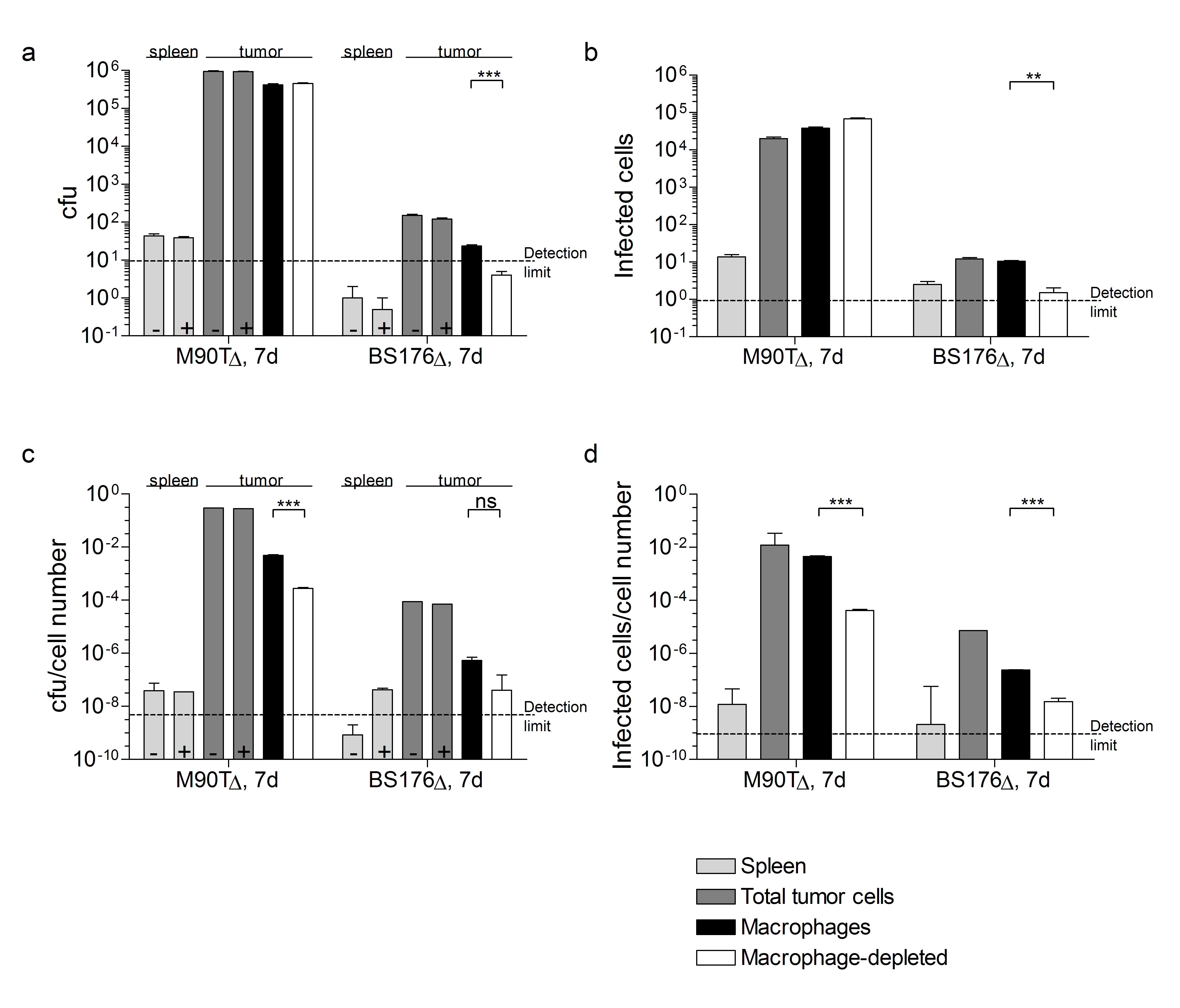 |

7 days after i.v. infection, *M90TΔaroA* predominantly resides within macrophages of spontaneous tumors (5 fold difference compared to macrophage-depleted fractions). The non-invasive *BS176∆aroA* strain is still present in the tumors in very low numbers and also found predominantly in macrophages. *M90TΔaroA* is capable of inducing caspase-1 processing and apoptosis in comparison to the non-virulent *BS176∆aroA* strain. All results shown are mean ± SD (n = 4); ** P < 0.01, *** P < 0.001, Student`s t-test.

| Supporting information Fig. S 8:  *Shigella flexneri M90TΔaroA*, but not *Shigella flexneri BS176ΔaroA* induced apoptosis in 4T1-induced tumor tissue 6h and 7d *p.i*.  *Terminal Deoxynucleotidyltransferase-Mediated dUTP-Biotin Nick End Labeling Assay*. Paraffin-embedded sections of tumor tissue isolated from 6 h and 7 d *S. flexneri M90TΔaroA* and *BS176ΔaroA*-infected 4T1-induced tumor-bearing Balb/c mice were stained according to the Dead End Fluorometric TUNEL System instructions (Promega). |
| --- |
| 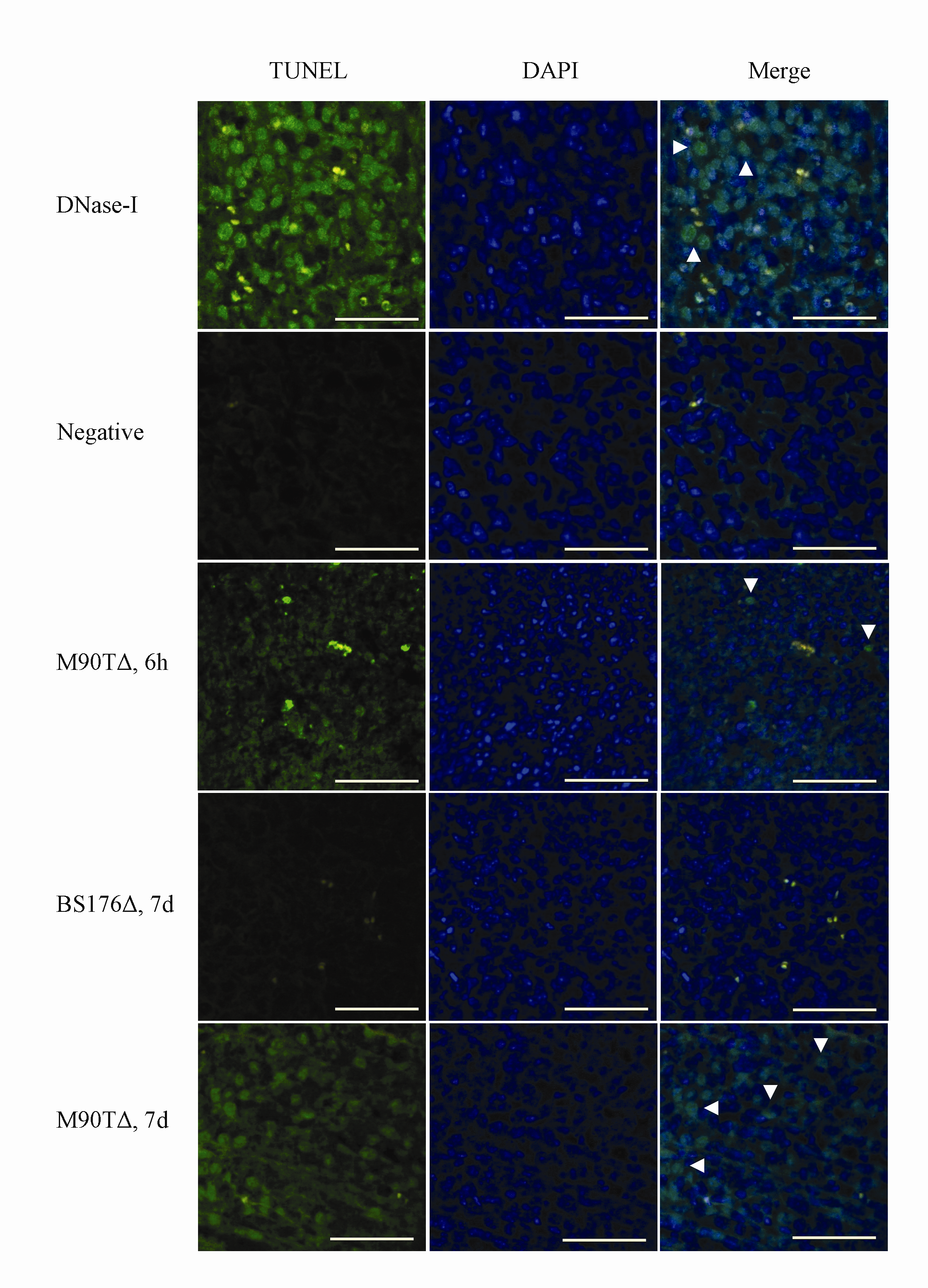 |
| Paraffin embedded sections of tumor tissue isolated from tumor-bearing Balb/c mice uninfected or infected with *BS176Δ* or *M90TΔ* were stained with a TUNEL protocol detecting apoptotic cells (green dots, left panels) and DAPI (blue dots, middle panels). Fragmented nuclei are exemplified with white arrowheads. Scale bars: 50 µm. BS176Δ: *Shigella flexneri BS176ΔaroA*; M90TΔ: *Shigella flexneri*. *M90TΔaroA*. |

| Supporting information Fig. S 9:  TAM number is dependent on tumor volume. To determine the number of TAMs in 4T1-induced tumors flow cytometer analysis were performed. After different time points post tumor cell inoculation tumors were removed from Balb/c mice and TAMs were separated by MACS®. Subsequently the total amount of TAMs was determined by flow cytometer via staining for F4/80 and CD11b. |
| --- |
| 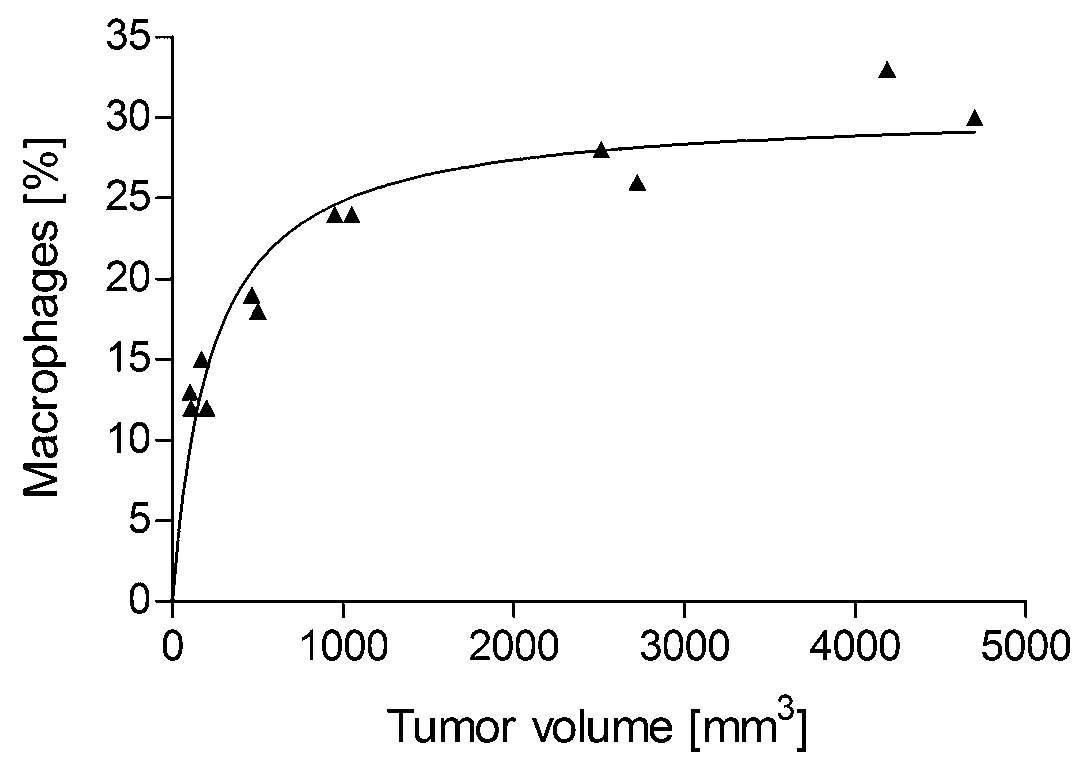 |

| Supporting information:Fig. S 10:  *M90TΔaroA* predominantly targets TAMs isolated from human cancer ascites cells in an *ex vivo* infection experiment.  Ovarian cancer ascites contains both adherent cancer (adh.) and non-adherent (susp.) myeloid cells. Tumor cells were separated and TAMs were isolated as described before. The different cell fractions isolated from an ovarian cancer patient were infected *ex vivo* with wt *S. flexneri M90T, S. flexneri M90TΔaroA* and *S. flexneri BS176ΔaroA*. After 1 h of infection with a MOI of 100, cells were incubated for 1 h with 300 µg/ml gentamicin. Afterwards, 50 µg/ml gentamicin were used. 2 h *p.i.* cells were harvested to determine CFU or were prepared for Western Blot analysis. All results shown are mean ± SD of three samples. |
| --- |
| 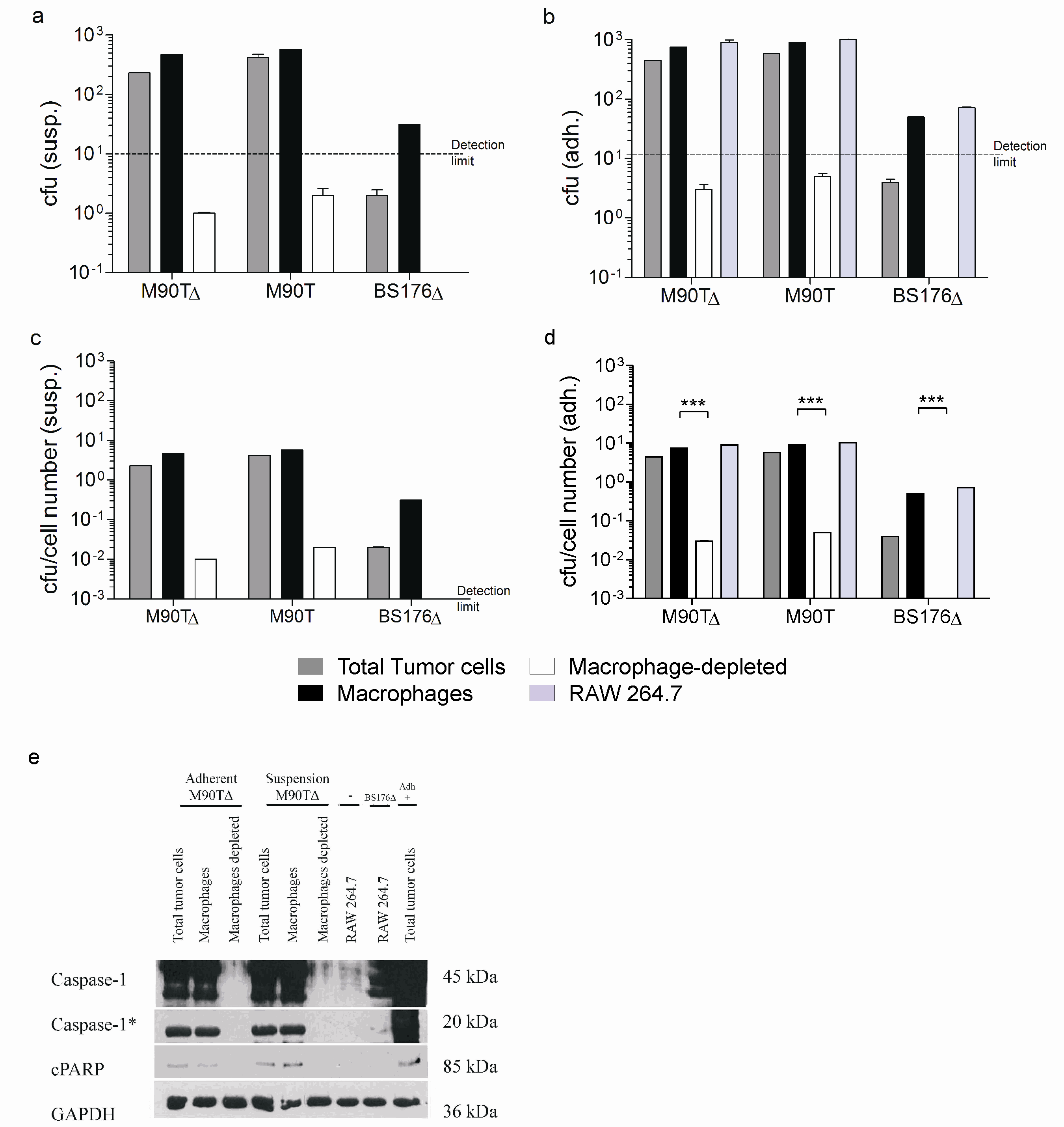 |

| Supporting information Fig. S 11:  *M90TΔaroA* infects 4T1 cells *in vitro*  1 x 10^6^ 4T1 cells (a, c) and RAW 264.7 macrophages (b, d) were infected *in vivo* with wt *S. flexneri M90T, S. flexneri M90TΔaroA* and *S. flexneri BS176ΔaroA*. After 1 h of infection with a MOI of 100, cells were incubated for 1 h with 300 µg/ml gentamicin. After 1h, 3h and 10h *p.i.* cells were harvested to determine CFU or were stained with Trypanblue dye to determine the ratio of dead and alive cells. All results shown are mean ± SD of two samples. |
| --- |
| 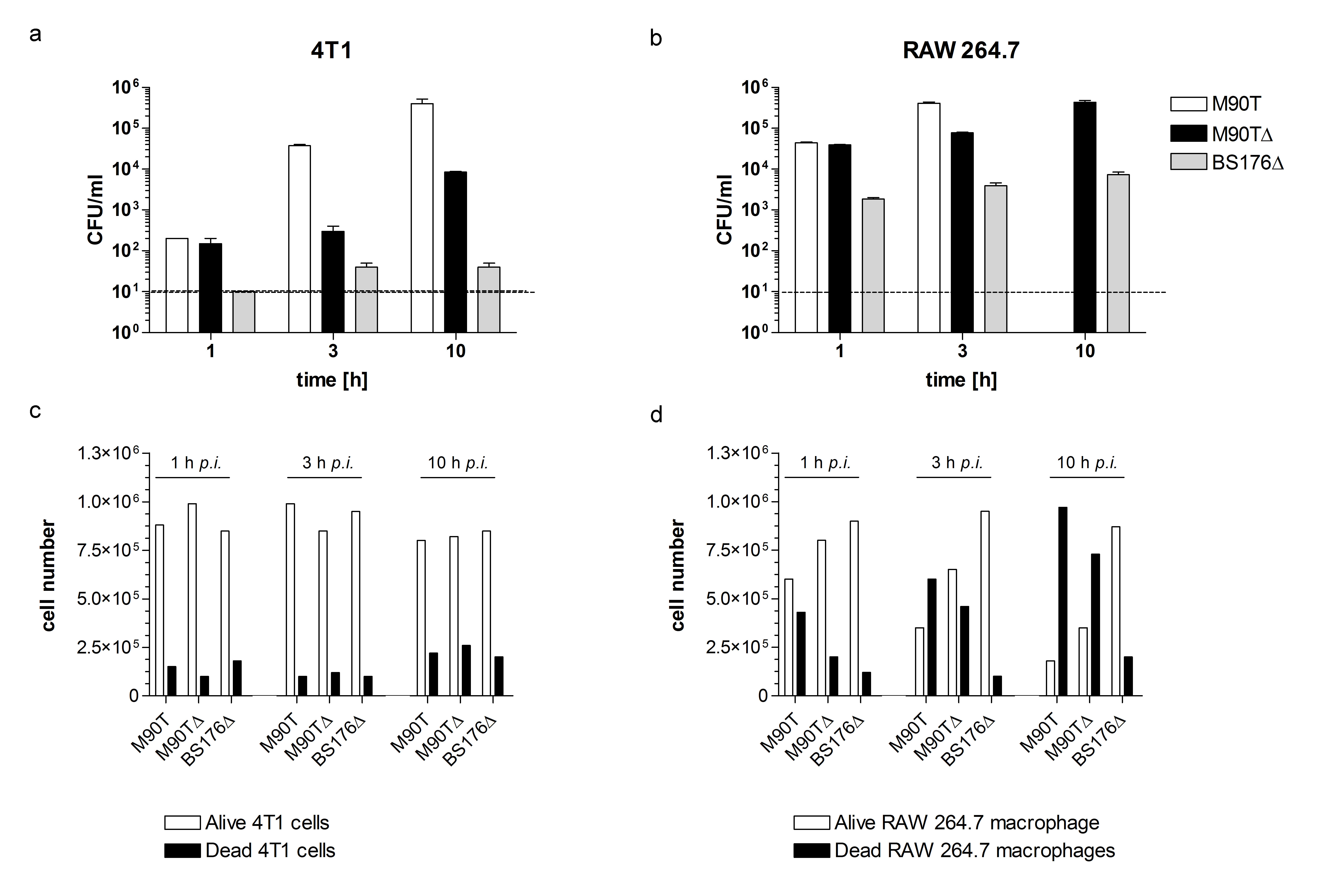 |
